# Plasma Zonulin as a Biomarker of Divergent Channels Latency in Systemic Lupus Erythematosus?

**DOI:** 10.3390/healthcare14142086

**Published:** 2026-07-12

**Authors:** Cristian-Mihai Ilie, Cătălina-Anamaria Boromiz, Denise-Ani Mardale, Irina Anna-Maria Stoian, Marilena Gilca, Dorin Dragos

**Affiliations:** 1 Department of Functional Sciences I/Biochemistry, Carol Davila University of Medicine and Pharmacy, 050474 Bucharest, Romania; cristian-mihai.ilie@rez.umfcd.ro (C.-M.I.); irina.stoian@umfcd.ro (I.A.-M.S.); 2 Department of Rheumatology and Internal Medicine, Sfânta Maria Clinical Hospital Bucharest, 011172 Bucharest, Romania; catalinaboromiz@gmail.com (C.-A.B.); mardaledenisse@gmail.com (D.-A.M.); 3 Department of Rheumatology, Carol Davila University of Medicine and Pharmacy, 011172 Bucharest, Romania; 4 Department of Medical Semiology, Faculty of Medicine, Carol Davila University of Medicine and Pharmacy, 020021 Bucharest, Romania; dorin.dragos@umfcd.ro; 5 1st Internal Medicine Clinic, University Emergency Hospital Bucharest, Carol Davila University of Medicine and Pharmacy, 050098 Bucharest, Romania

**Keywords:** divergent channels, sinew channels, zonulin, (1→3)-β-D-glucan, intestinal permeability, systemic lupus erythematosus, joints, Chinese Medicine

## Abstract

**Highlights:**

**What are the main findings?**

- Patients with systemic lupus erythematosus (SLE) had higher plasma zonulin and (1→3)-β-D-glucan levels than controls, suggesting impaired intestinal barrier function, and fungal translocation or proliferation.
- Similar levels of plasma zonulin between patients with large joints involvement and controls may be an expression of the pathogen latency in large joints.

**What are the implications of the main findings?**

- Plasma zonulin may be a candidate biomarker of divergent channels latency in SLE.

**Abstract:**

**Background:** Systemic lupus erythematosus (SLE) is an autoimmune disorder with a chronic intermittent course, a characteristic that suggests an involvement of divergent channels. According to Chinese medicine, divergent channels divert the pathogens to joints, where they are held transiently in a latent state, in order to keep them away from internal organs. The purpose of the present work is to determine whether, in SLE subjects, the levels of certain markers of gut leakage (zonulin), fungal translocation or proliferation of [(1→3)-β-D-glucan], and protective mechanisms (FGF21) are associated with the type of organ involvement or with the type of joint involvement (small vs. large joints) and to explore potential connections with the Chinese medicine perspective on SLE, conceptualized as pathology of the divergent channels. **Methods:** The study had a cross-sectional design and included an SLE group (*n* = 33) and a control group (*n* = 24) of subjects. Plasma levels of zonulin, (1→3)-β-D-glucan, and FGF21 were measured using indirect ELISA. **Results:** Despite the fact that SLE patients exhibited higher plasma zonulin levels when compared to controls [4.709 (1.996–10.426), *n* = 33 vs. 1.973 (1.371–3.05925), *n* = 24, *p* = 0.004], [median (IQR)], there was no difference in plasma zonulin levels between SLE patients with large joint involvement and controls. Plasma (1→3)-β-D-glucan was also higher in the SLE group than in controls [119.444 (82.746–277.66), *n* = 33 vs. 77.893 (47.524–124.7495), *n* = 24, *p* = 0.01]. Cutaneous involvement was significantly more frequent in patients with large joint (*p* = 0.03) and small joint (*p* = 0.006) involvement than in those without articular involvement. **Conclusions:** Plasma zonulin may be correlated with the degree of SLE latency in joints (divergent channels activation). The engagement of divergent channels, reflected by articular involvement, appears to be associated with a greater propensity for sinew channel activation as a potential mechanism of pathogen evacuation.

## 1. Introduction

Systemic lupus erythematosus (SLE) is a chronic multisystemic autoimmune disease that may affect the joints, skin, kidneys, lungs, nervous system, hematologic system, serous membranes, and heart [1,2]. The most frequent clinical manifestation in SLE is the musculoskeletal: up to 95% of patients experience joint symptoms [3]. Lupus arthritis predominantly involves the small joints of the hands, wrists, and knees, and also the hip joint [4].

Although its precise etiology remains incompletely understood, SLE is considered to result from a complex multifactorial interplay between hormonal, genetic, and environmental factors [5]. Also, disruption of gut microbiome balance and compromised intestinal barrier have become increasingly relevant in SLE pathogenesis. Gut microbiome dysbiosis is frequent among patients with SLE and may contribute to impaired gut barrier integrity with microbial translocation, increased exposure to microbial antigens, and consequent immune system activation [6]. This mechanism is supported by an in vitro study, which showed that microbiota isolated from stool samples of SLE patients increased lymphocyte activation and their differentiation into Th17 cells [7].

Available evidence suggests a bidirectional relationship between intestinal barrier dysfunction and SLE. On one hand, increased intestinal permeability may exacerbate SLE activity by promoting the systemic translocation of bacterial and fungal products, thereby activating innate immune cells through TLR4 and Dectin-1 signaling pathways. On the other hand, SLE may contribute to impaired gut permeability through immune complex deposition-mediated vascular injury and the effects of commonly used therapies, including nonsteroidal anti-inflammatory drugs and immunomodulatory agents [8].

Dormant microorganisms can migrate from gut mucosae to joints and other distant sites through blood (by using macrophages as Trojan horses [9] by hiding themselves within autophagosomes [10]) or through lymphatics [11]. Persistent microbes (including those from gut microbiota) in bone-marrow mesenchymal stem (BMMS) cells from the epiphyses of joints were suggested to be contributing pathogenic factors to chronic inflammatory joint diseases [12]. Scientists have suggested that these dormant infections may lead to a kind of trained immunity in daughter BMMS cells in joints that lasts even after the microbes are cleared [12].

(1,3)-β-D-Glucan (1,3-BDG), a cell wall constituent of several fungal species (including Candida, Aspergillus, and Pneumocystis), is released during fungal proliferation and represents a biomarker of fungal translocation or proliferation [13,14]. Several studies have reported elevated beta-glucanemia in lupus animal models, attributed either to enhanced gut permeability or increased fungal burden within the gut microbiota, suggesting a possible impact and worsening effect in lupus progression [15,16]. Synergy of β-glucanemia and endotoxemia exacerbated lupus activity in animal models by induction of colonic neutrophil extracellular traps that facilitate microbial gut translocation [17] and by enhancement of the macrophage cytokine production and inflammatory state [18].

There are also various protective mechanisms (e.g., farnesoid X receptor pathway activation, leading to increased fibroblast growth factors (FGFs) FGF21, and FGF19 levels), which regulate microbiota diversity, prevent gut dysbiosis and intestinal tight-junction damage, and maintain the intestinal barrier [19]. Recent studies have demonstrated that the FGF21–bile acids–gut microbiota–short-chain fatty acids axis mediates these beneficial effects across a variety of pathological conditions [19,20,21]. FGF21 modulates the profile of fecal bile acids (which exert antimicrobial and complex signaling activities) and stimulates gut microbiota production of short-chain fatty acids (which represent the most important “fuel” for the intestinal epithelial cells, inhibitors of harmful bacteria proliferation, and protective factors against intestinal damage) [22]. FGF21 was also suggested to delay lupus progression in an animal model [23]. However, one human study found a positive correlation of FGF21 with global disease activity, and it was increased in subjects with renal involvement [24]. Given the limited number of studies investigating the potential role of FGF21 in SLE patients, exploring this direction deserves attention.

Zonulin is a protein that acts as a regulator of intestinal tight junctions, and its increased serum levels are associated with increased intestinal permeability [25]. In one of our previous studies, besides the fact that we have found that SLE patients have higher levels of plasma zonulin than controls, we have also found that SLE patients without clinical joint involvement had unexpectedly higher plasma levels of zonulin than those with this feature [26]. We suggested that this intriguing result may be at least partially explained on the basis of a bidirectional gut–joint iteropathy of lymphocytes (bidirectional migration of lymphocytes between the digestive tract and joints within the lymphoid network), so that the inflammatory “signature” emerges either at the intestinal or articular level, reflecting the preferential localization and accumulation of lymphocytes within distinct compartments of the gut–joint axis [26].

Supporting the gut–joint axis concept, a murine model of spondylarthritis demonstrated that intraepithelial T cell lymphocytes from the distal colon can migrate to the Achilles tendon enthesis and promote local inflammation through IL-17 and TNF production [27].

At present, there is no direct evidence of lymphocyte trafficking from the joints to the gut; however, this possibility cannot be excluded, given the existence of general immunological mechanisms of lymphocyte homing to the gut, especially in inflammatory conditions [28]. For instance, lymphocytes expressing the integrin α4β7 on their surface (which interact with the cell adhesion molecule MAdCAM-1 expressed in the gut) and the chemokine receptor CCR9 (which recognizes CCL25, constitutively expressed by intestinal epithelial cells) are imprinted with gut tropism [29,30,31].

These previous findings, which have not yet been sufficiently explained from a biomedical science perspective, prompted us to further investigate them, also through the lens of Classical Chinese Medicine (CCM).

### Classical Chinese Medicine Perspective on Systemic Lupus Erythematosus

Several features of SLE, including the chronic course characterized by a pattern of episodic flare-ups and remissions, as well as its autoimmune pathogenesis [2], suggest, according to literature on Chinese Medicine, an involvement of divergent channels, which represents one of the five types of energetic channels, together with sinew channels, luò vessels, primary channels, and extraordinary channels [32,33]. According to these resources, the essential role of divergent channels, as the last protection barrier, is to divert the pathogenic factor (xié qì) away from the internal organs (zàng fǔ) to avoid their disturbances and structural damage, and to send it to the joints, where it is held latent, transiently trapped as long as the essence (jīng qì) is available, even at the expense of other resources (e.g., Blood-xuè, Fluids- jīn yè) [32,33]. As long as the pathogenic factor is hidden in the joints, the disease enters a so-called “latency phase’’, divergent channels functioning as a buffer between the external and the internal sites.

The concept of pathogenic factors (xié qì) in Chinese medicine covers a widespread range of factors that have in common the property of challenging the body’s homeostatic mechanisms: the climatic factors, imbalanced diet, microbes, environmental pollutants or xenobiotics, and psycho-emotional stress. Interestingly, accumulating evidence suggests that nearly all of these Chinese pathogenic factors (CPFs) can induce dysbiosis and compromise the intestinal barrier (Table 1).

The consequent gut leakage, observed in SLE [26,52], may facilitate the translocation of gut microbial components (e.g., (1→3)-β-D-glucan) that may be included in the category of CPFs into systemic circulation, promoting inflammation in distant organs and autoimmunity [52,53]. In CCM, autoimmune self-destructive tissue processes are understood as the consequences of Heat (analogous to the calor aspect observed in inflammation) generated during the “efforts” of protective energy (Wèi Qì) to counteract pathogenic influences, with the resulting internal Heat contributing to tissue damages [32,33].

**Figure 1:**
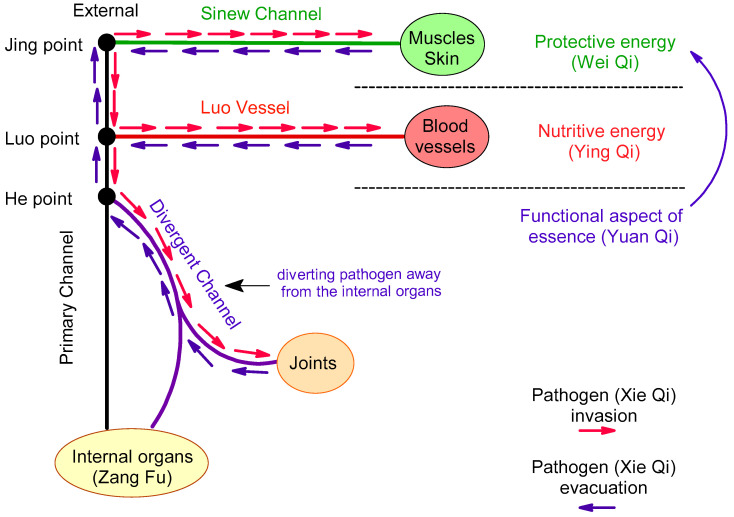
Pathways of invasion and evacuation of pathogens (Xie Qi) and the role of divergent channels as the last protective barrier of internal organs.

Interestingly, within the framework of CCM, the pathogenesis of disease is conceptualized as the progressive invasion of the CPF through distinct energetic layers, occurring concomitantly with the failure of the energetic bodily defense mechanisms—analogous to the previously described translocation of microbes or microbial components from a compromised intestinal barrier to distant sites. This process is understood to advance sequentially from the most superficial or protective level (Wèi layer), through the intermediate or nutritive level (Yíng layer), and ultimately to the deepest or constitutional level (Yuán layer), each of them characterized by a specific type of energy (Qi) (Figure 1).

Each energetic layer has certain correspondences with the system of channels, fluids, and structural constituents [32,54] (Table 2).

The divergent channels are large branches (divergences or extensions) of the primary channels that connect external to internal sites [57]. They function in tandem with the sinew channels, supporting them by mobilizing Yuán Qì from a deeper level (Yuán layer) towards the superficial level (Wèi layer), which is mostly specialized in expelling the pathogen [54]. Interestingly, according to immunogenetics, there is a strong coordination between the genetic level (corresponding to the Yuán layer) and immune processes (corresponding to the Wèi layer) [56]. For instance, immune system function under circadian rhythms coordinated by clock genes (e.g., genes controlling diurnal rhythms in inflammatory monocyte numbers [58] and diurnal expression of proinflammatory cytokines [59]), similarly to Wèi Qi, which also has a circadian rhythm, circulating 50 times daily (25 times during the daytime, at the surface of the body, and 25 times during the night, in the body’s internal part) [60]. Moreover, scientists have suggested that these clock genes, which initiate a rhythmic inflammatory signal, might even be responsible for the diurnal variation in the symptoms and severity of inflammatory arthritis [61].

Regarding the articular involvement in SLE within the framework of CCM, it is particularly noteworthy that all divergent channels are connected with the large joints (hips, knees, shoulders, and sacroiliac joints), almost all starting at the level of Hé points located in the proximity of these large joints [33,54]. Thus, the concept emerged of stratifying SLE patients into subgroups according to the presence or absence of articular involvement and its type (involving large versus small joints), as well as evaluating potential differences between these subgroups.

The aim of this study is to determine whether, in patients with SLE, the levels of certain markers of gut leakage (zonulin), microbial translocation (1,3-BDG), and protective mechanisms (FGF21) are associated (1) with the type of organ (cutaneous vs. articular vs. deep organ) involvement and (2) with the type of joint involvement (small joints vs. large joints vs. no joint involvement) and to find a potential connection with the theory of pathogenesis from CCM.

## 2. Materials and Methods

### 2.1. Study Design and Participant Selection

We conducted a cross-sectional study at “Sfânta Maria” Clinical Hospital, Bucharest, Romania, between January 2024 and May 2024. The study population comprised 57 participants, including 24 healthy volunteers and 33 patients diagnosed with SLE, who fulfilled the 2019 ACR/EULAR classification criteria for SLE [62]. All participants were adults of Romanian nationality. Healthy controls were recruited from individuals without a history of autoimmune or autoinflammatory disease. Exclusion criteria applied for both cohorts included recent infection within 3 months, history of invasive fungal infection, diarrhea or a history of diarrhea within 3 months, ongoing malignancy, or the presence of additional inflammatory, autoimmune, or autoinflammatory disease.

Each participant attended a single study visit. For patients with SLE, the study visit was scheduled to coincide with a routine follow-up assessment at the hospital, whereas healthy controls were evaluated during a single dedicated study visit. All eligible individuals were informed about the study objectives and enrolled after providing written informed consent.

### 2.2. Study Variables and Sample Collection

The biomarkers of interest were plasma zonulin, 1,3-BDG, and fibroblast growth factor 21 (FGF21). These analytes were quantified from blood samples collected specifically for the study using enzyme-linked immunosorbent assay (ELISA). Additional demographic, anthropometric, clinical parameters, and routine laboratory parameters were retrieved from the patients’ medical records. These included sex, age, urban/rural background, body mass index (BMI), disease duration since symptom onset, disease duration since diagnosis, and disease activity assessed using the SLE Disease Activity Index (SLEDAI) score [63], organ damage level assessed using the Systemic Lupus International Collaborating Clinics/American College of Rheumatology (SLICC/ACR) Damage Index [64], and medication profiles.

As matching parameters, demographic (sex, urban/rural background) and anthropometric [age, body mass index (BMI)] variables were taken into account.

Renal and neurological involvement were considered deep organ involvement, as in the natural history of SLE, these complications may be life-threatening.

In addition to the standard clinical and laboratory assessment, a supplementary 4 mL blood sample was collected into EDTA K2 tubes (BD Vacutainer, Franklin Lakes, New Jersey, USA) after a 12 h overnight fast. Samples were processed by centrifugation at 10,000× *g* for 15 min, after which the plasma fraction was transferred into separate aliquots and stored at −80 ֯C until laboratory assessment of the selected biomarkers.

### 2.3. Biomarker Assessment

Plasma concentrations of zonulin, 1,3-BDG, and FGF21 were determined using commercially available enzyme-linked immunosorbent assay kits in accordance with the manufacturers’ instructions. Zonulin was measured using the Human Zonulin ELISA 96T kit (Cusabio, Wuhan, China), whereas 1,3-BDG and FGF21 were quantified using the Human BDG (beta-D-glucan) ELISA 96T kit (FineTest, Wuhan, China) and the Human FGF21 (Fibroblast Growth Factor 21) ELISA 96T kit (FineTest, Wuhan, China). All samples were analyzed in duplicate, and optical density was measured using a Mindray MR-96A microplate reader (Shenzhen Mindray Bio-Medical Electronics Co., Ltd., Shenzhen, China). Mean values from duplicate determinations were used for statistical analysis. The inter-assay coefficients of variation were within the acceptable limits specified by the manufacturers.

### 2.4. Statistical Analysis

All the statistical computations and graphical representations were performed using the R language and environment for statistical computing and graphics version 4.6.0 (R Project for Statistical Computing). Packages car, tidyverse, ggplot2, ggpubr, mosaic, mediation, dplyr, fmsb, and reporttools have been used.

The Shapiro–Wilk test was conducted to determine whether the numerical (continuous) data were normally distributed. The association of dichotomous (binary) categorical variables with normally and non-normally distributed numerical variables was explored by means of Student’s t test and Mann–Whitney test/Wilcoxon rank sum test, respectively.

The Kruskal–Wallis rank sum test was employed to investigate the relationship between numerical (continuous) variables (such as zonulin plasma level) and polytomous (with multiple levels) categorical variables (such as the type of joint involvement: small vs. large vs. no joint involvement).

Pairwise comparisons using the Mann–Whitney test/Wilcoxon rank sum test with *p* value adjustment by the Bonferroni method were thereafter performed to establish which are the actual statistically significant differences among the various levels of the polytomous categorical variable.

Fisher’s exact test was employed to investigate the relationship between a dichotomous categorical variable [such as cutaneous involvement or deep organ (renal or neurological) involvement in LES patients] and a polytomous categorical variable (namely, the type of joint involvement: small vs. large vs. no joint involvement). Knee, hip, shoulder, spine, and sacroiliac joints were included in the category of large joints, while hand joints were considered small joints.

In the case of statistically significant results, pairwise comparisons using Fisher’s exact test with *p* value adjustment by the Bonferroni method were thereafter performed to establish which levels of the polytomous categorical variable were actually statistically significantly associated with the dichotomous categorical variable.

A group of healthy individuals was chosen, matched with the SLE patients in terms of anthropological [sex, age, body mass index (BMI)] and demographic variables [background/residency (urban vs. rural)] (Table 3). Fisher’s exact test was employed to prove the absence of significant differences between the two groups regarding sex and background/residency. The Shapiro–Wilk test showed that both age and BMI had a normal distribution; therefore, the mean and standard deviation were used to characterize these two parameters, and Student’s t test was employed to establish whether there were/to demonstrate the absence of significant differences between the two groups.

The results were considered statistically significant if the *p*-value < 0.05.

## 3. Results

Shapiro–Wilk test demonstrated that, among numerical variables, age and BMI were normally distributed, while FGF21, zonulin, and 1,3-BDG were not.

### 3.1. Baseline Clinical, Demographic, and Anthropometric Characteristics

The demographic and anthropometric characteristics of the participants, including age, sex, and urban/rural background, are summarized in Table 3.

The main clinical features of the SLE group are summarized in Table 4.

The medication profile of the SLE group reflected heterogeneous therapeutic exposure. Corticosteroids were used in 23 patients. Hydroxychloroquine was administered in 24 patients, of whom 1 received it in combination with Methotrexate, 3 with Mycophenolate mofetil, and 7 with Azathioprine. Belimumab was used in five patients, including four in combination with hydroxychloroquine and one in combination with Mycophenolate mofetil.

### 3.2. Biomarkers Profile and Routine Laboratory Findings of the SLE Group

The main laboratory parameters of the SLE group are summarized in Table 5.

### 3.3. Comparison of Biomarker Levels Between SLE and Controls

**Figure 2:**
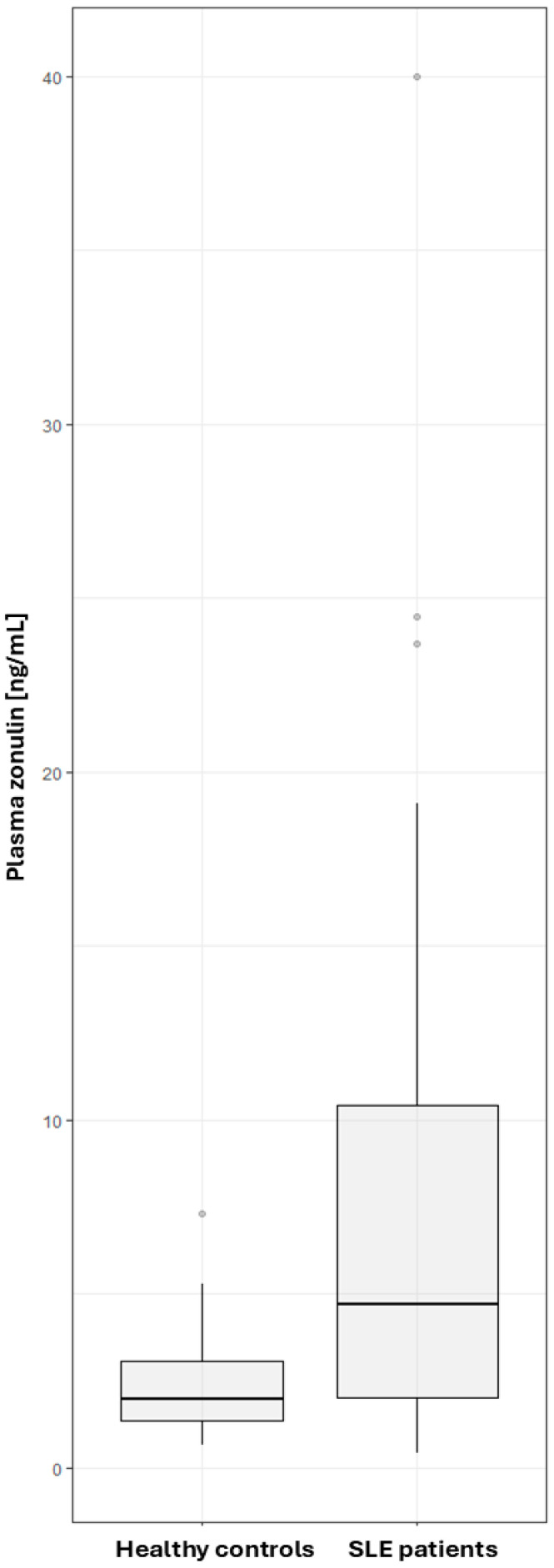
Plasma zonulin (ng/mL) estimated as median (IQR- interquartile range) in SLE patients [4.709 (1.996–10.426), *n* = 33] vs. Healthy controls [1.973 (1.371–3.05925), *n* = 24], median difference (95% confidence interval) = −2.251 (−4.530 to −0.625), W statistics = 220, *p* = 0.004 by Mann-Withney-U-test; The dots (•) represent the outliers.

**Figure 3:**
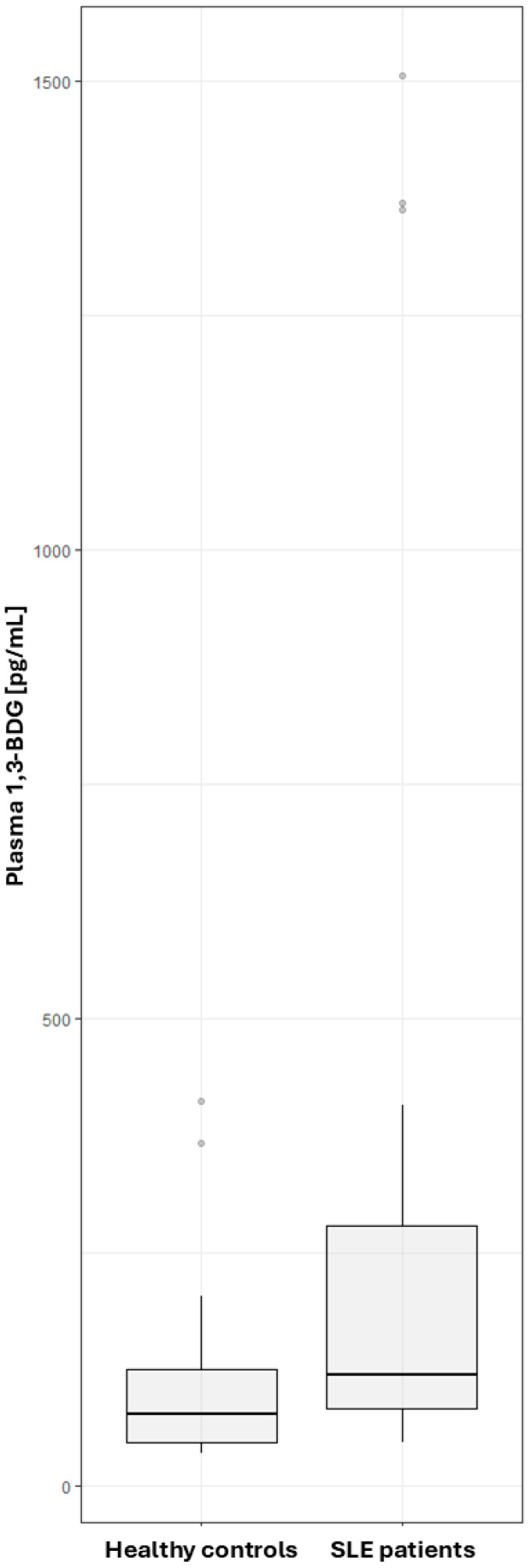
Plasma 1,3-BDG (pg/mL) estimated as median (IQR- interquartile range) in SLE patients [119.444 (82.746–277.66), *n* = 33] vs. Healthy controls [77.893 (47.524–124.7495), *n* = 24], median difference (95% confidence interval) = −39.902 (−81.398 to −10.192), W statistics = 244, *p* = 0.01 by Mann-Withney-U-test); The dots (•) represent the outliers.

The comparison between the study group and the control group regarding the three markers (Mann–Whitney test) was done by means of the Mann–Whitney test (Table 6). The statistically significant results are displayed in Figure 2 and Figure 3.

### 3.4. Analysis of Biomarkers in Relation to Clinical Involvement

**Figure 4:**
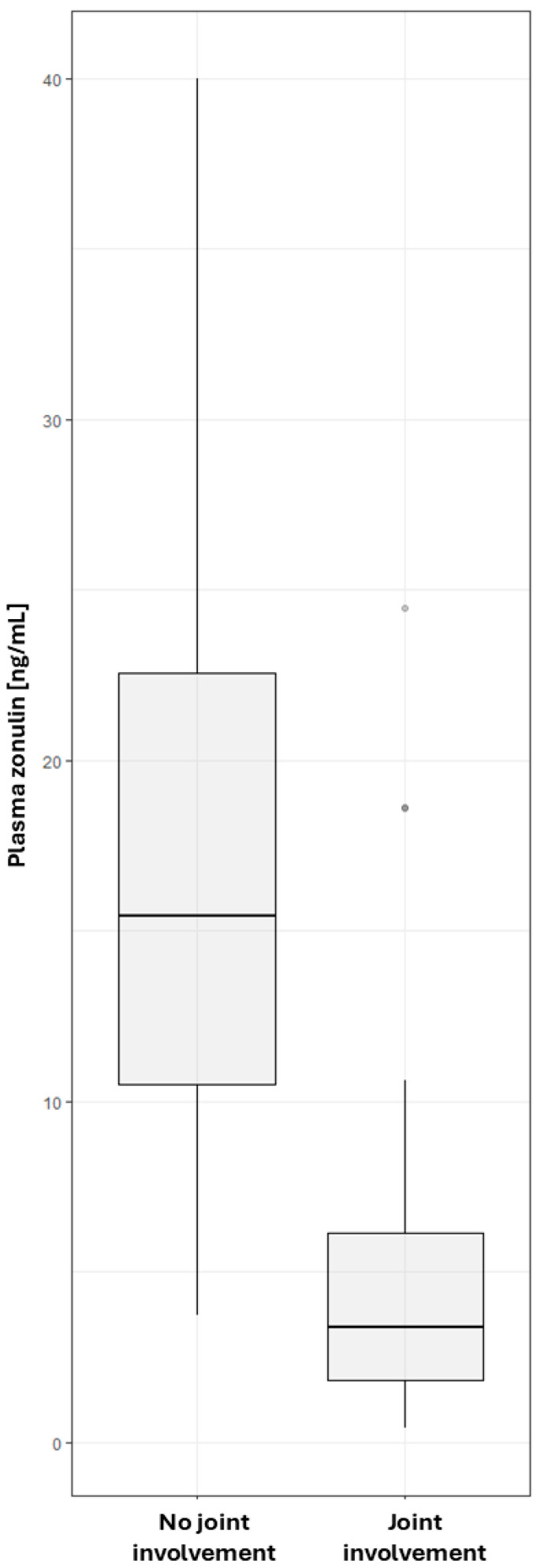
Plasma zonulin (ng/mL) estimated as median (IQR- interquartile range) in SLE patients without joint involvement [15.436 (10.49–22.54575), *n* = 6] compared with SLE patients with joint involvement [3.363 (1.816–6.1405), *n* = 27]; median difference (95% confidence interval) = 9.830 (2.872 to 20.847), W statistics = 139, *p* = 0.007, by Mann–Whitney-U-test); The dots (•) represent the outliers.

The association between each of the three markers (Mann–Whitney test) and the type of involvement was explored by means of the Mann–Whitney test (Table 7). The relationship between plasma zonulin and articular involvement is represented in Figure 4.

**Figure 5:**
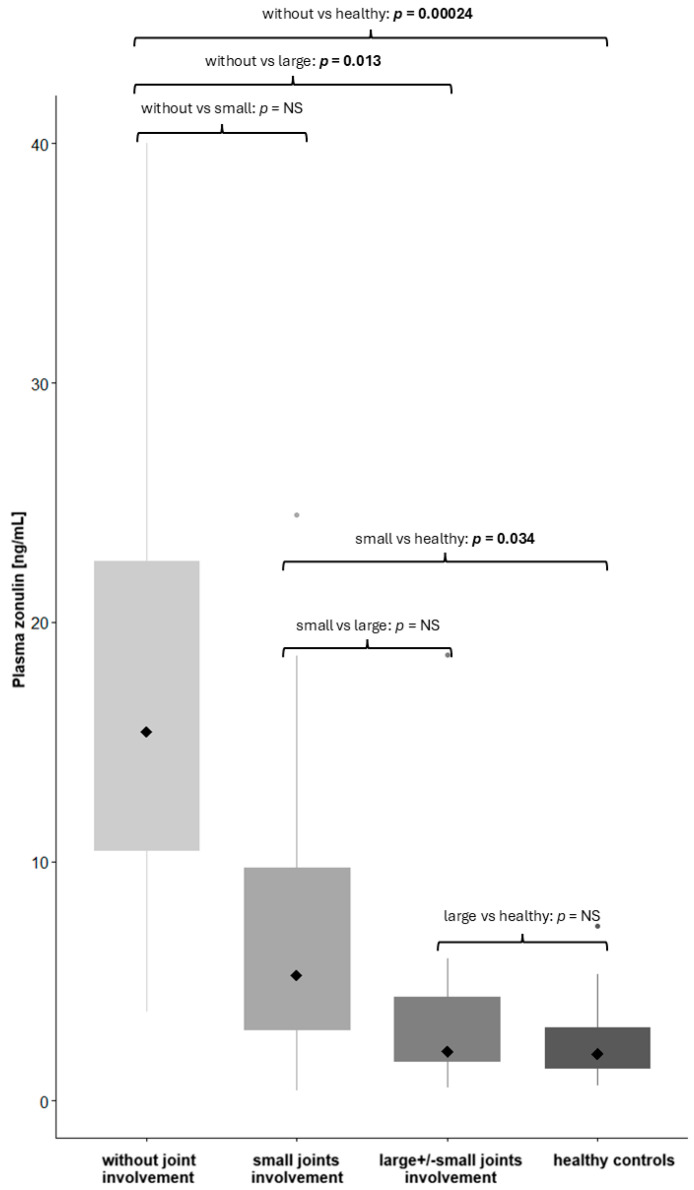
Plasma zonulin (ng/mL) estimated as median (IQR- interquartile range) in SLE patients without articular involvement [15.436 (10.49–22.54575), *n* = 6] compared with SLE patients with small joint involvement [5.2525 (2.973–9.7515), *n* = 14], with SLE patients with large joint involvement with or without concomitant small joint involvement [2.061 (1.672–4.35), *n* = 13], and with healthy controls [1.973 (1.371–3.05925), *n* = 24]; *p* = 0.00046, Kruskal–Wallis test; The diamond symbol (◆) inside the boxes indicates the median.

The Kruskal–Wallis rank sum test demonstrated a statistically significant association between zonulin plasma level and the type of joint involvement [small vs. large (±small) vs. no joint involvement] in SLE patients. For reference, the zonulin plasma level in healthy individuals was also included in the computation: a *p*-value = 0.00046, corresponding to a Kruskal–Wallis chi-squared of 17.908 with 3 degrees of freedom. The results of the pairwise comparisons by means of the Mann–Whitney test with *p* value adjustment by the Bonferroni method are displayed in Table 8 and illustrated in Figure 5.

**Figure 6:**
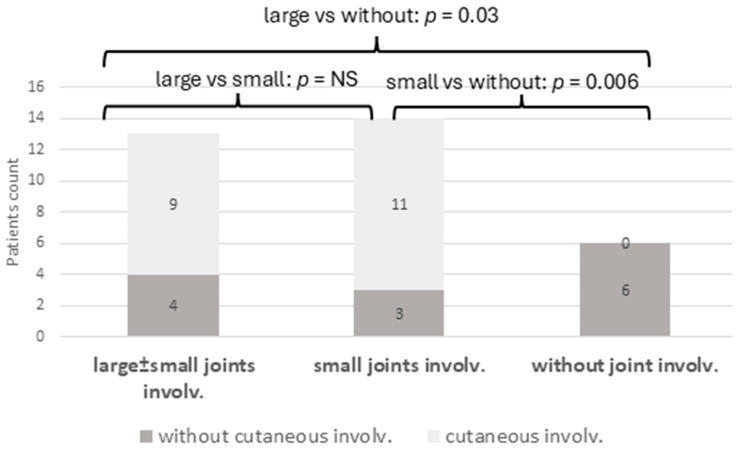
Association between cutaneous involvement and the type of joint involvement in SLE patients—pairwise comparisons using Fisher’s exact test with *p* value adjustment by the Bonferroni method.

Fisher’s exact test demonstrated no association between deep organ (renal or neurological) involvement in LES patients and the type of joint involvement (small vs. large vs. no joint involvement) (*p* value = 0.3). However, there was a statistically significant association between cutaneous involvement and the type of joint involvement (*p* value = 0.003). Pairwise comparisons using Fisher’s exact test with *p* value adjustment by the Bonferroni method were thereafter performed to establish which levels of the polytomous categorical variable were actually statistically significantly associated with the dichotomous categorical variable (Figure 6, Table 9).

Regarding the correlation between the three markers and disease duration, the Spearman test was used with *p* value correction by the Bonferroni method—none of the results was statistically significant (Table 10).

Regarding the correlation between the three markers and SLEDAI, the Spearman test was used again with *p* value correction by the Bonferroni method—none of the results was statistically significant (Table 11).

## 4. Discussion

SLE has complex multifactorial pathogenesis, with increasing evidence supporting a contributory role for gut microbiome dysbiosis and impaired intestinal barrier integrity.

One of the novelties of the present study is the concomitant assessment of zonulin, 1,3-BDG, and FGF21 in SLE patients, allowing a more integrated evaluation of epithelial barrier dysfunction, fungal translocation or proliferation, and potential protective mechanisms in SLE.

Higher plasma levels of zonulin in the SLE group than in controls suggest the presence of impaired intestinal barrier integrity, a finding that is consistent with the results reported by other researchers [65].

Higher levels of 1,3-BDG in the SLE group may signify either local gut fungal proliferation or fungal translocation. Since the exclusion criteria in the SLE group included a history of infection or diarrhea within 3 months and a history of invasive fungal infection, the most probable explanation for the increased 1,3-BDG level is fungal translocation. Nevertheless, further studies involving direct measurements of gut fungal burden are required to elucidate the precise cause of these elevated values in the context of SLE.

A previous study found that the 1,3-BDG level was higher in SLE human subjects with active lupus nephritis than in those with an inactive condition [18], suggesting that 1,3-BDG might affect disease severity in the case of renal involvement. Our results showed no statistical difference between subjects with deep organ (renal/neurological) involvement and those without, suggesting that 1,3-BDG may not be associated with this type of internal organ damage.

The most notable finding of our study was that there was no significant statistical difference in plasma zonulin levels between SLE patients with large joint involvement and controls, while there is an increasing trend of these values in the following series: group 1 (large joint involvement) < group 2 (small joint involvement) < group 3 (no joint involvement). A potential explanation is that large joints, owing to their greater synovial surface area and larger volume of synovial fluid, may accumulate higher levels of environmental toxins with pro-inflammatory properties. Although xenobiotics have been detected in joint tissues (e.g., heavy metals [66,67], microplastics [68,69]) or even selectively accumulated by synovial cells and chondrocytes (e.g., mercury) [70], no studies to date have compared the abundance of these compounds between large and small joints.

However, this subgroup analysis regarding the correlation between the type of articular involvement and plasma zonulin levels should be considered strictly exploratory, as it is underpowered given the small size of our sample. Moreover, as joint involvement is the most common clinical feature of SLE, present in up to 95% of patients [3], the subgroup of patients without joint involvement is inevitably small.

The direct correlation between the articular and skin involvements in both large and small joint groups is not unexpected, as musculoskeletal involvement is recognized as the most common clinical manifestation (affecting up to 95% of SLE patients), while the cutaneous is the second most frequent one (70–85% of SLE patients) [71]. The concomitant damage of skin and synovium in SLE may be interpreted as a result of the simultaneous local production of type I interferons, known to be key players in SLE pathogenesis, by both keratinocytes and synovial stromal cells, which share a common immunological program [72].

Regarding FGF21, this is a stress-inducible pleiotropic hormone secreted by the liver, which plays key regulatory roles in several metabolic processes (e.g., energy expenditure, glucose homeostasis), targeting mainly the adipocytes and neurons [73,74,75]. Significantly for our topic is that, besides its beneficial metabolic impact, FGF21 was also suggested to display some anti-inflammatory potential in lupus nephritis [23] and rheumatoid arthritis [76]. Nevertheless, our results, showing no statistical difference between FGF21 plasma levels in SLE subjects and controls, do not provide support for a protective role. At the same time, it does not exclude the possibility of a protective role against lupus nephritis, given that our study group comprised patients with and without renal involvement not confirmed as lupus nephritis with immune complex deposition through renal histopathology.

### 4.1. Classical Chinese Medicine Hypothesis

The topic of channel divergence was suggested to be a “benchmark of ambiguity” in the field of CCM, since Chapter 11 of the Divine Pivot (Líng Shū), the historical reference, is dedicated to their concise description [60] does not offer enough information about their therapeutics [77]. Nevertheless, there are some resurrected CCM resources, which approached in a detailed way the topic of divergent therapeutics, originating in the lectures of Jeffrey Yuen, a lineage holder of the 88th generation in the Jade Purity Yellow Emperor Lao Zi tradition (Yù Qīng Huáng Lǎo Pài) [32,78].

According to CCM theory, the pathogenesis of an autoimmune disease implies progressive penetration of the CPFs from the most superficial (Wèi layer) towards the deepest (Yuán layer) energetic layer. In the case of SLE, various types of antinuclear antibodies (e.g., anti-double-stranded (ds)DNA, anti- Sm nucleoprotein) are recognized as “quintessential biomarkers of SLE” [79] and also important players in SLE pathogenesis (via immune complexes with nuclear antigens, responsible for cell destruction, including genetic material degradation) [80]. Moreover, there are genetic pathogenic variants associated with SLE risk [81,82]. Within the conceptual framework of CCM, these molecular features, involving, to some extent, genetic-level processes—corresponding to the Yuán layer and the divergent channels—suggest that SLE is a disorder of the divergent channels.

During the evolution of a chronic disease involving divergent channels, symptomatic phases alternate with asymptomatic (or latency) phases when pathogenic factors are held latent in joints as long as the resources (Essence, Blood, Fluids, Qì, and Yáng) are available [33].

Elevated serum levels of 1,3-β-D-glucan observed in the SLE group may be interpreted either as a marker of increased CPFs burden within the gastrointestinal tract or, in the absence of overt fungal infection, as suggestive of translocation secondary to impaired intestinal barrier function and disruption of protective Wei Qi activity.

Zonulin levels directly reflect intestinal permeability (“gut leakage”) and indirectly indicate the extent of pathogen translocation through the gastrointestinal tract. This may correspond, within the CCM framework, to a dysfunction of the protective *Wei Qi* at the intestinal level and a potential involvement of the divergent channels, which are thought to redirect pathogenic factors toward the large joints as part of a systemic response aimed at preventing invasion of the internal organs (Zàng Fǔ).

Regarding the joint capacity to sequester the potential CPFs, there are several studies that showed that various types of CPFs might be bioaccumulated within the synovium or synovial fluid in case of exposure (e.g., heavy metals [83], microplastics [68]). The lymphatic drainage of synovial fluids plays an important role in normal synovium [84], and its failure was proposed to cause the progression of various rheumatological diseases (e.g., rheumatoid arthritis [85], osteoarthritis [86]). The abundance of the lymphatic network was associated with the activation phase of this drainage function in articular inflammation [87]. Interestingly, within the framework of CCM, divergent channels were compared with lymphatic vessels based on the following common characteristics:

1. Similarity in function: (a) the lymphatic system can evacuate pathogens from the blood circulation through immunosurveillance and immune responses [88], similarly with divergent channels, which can absorb CPFs from blood in case of previous barrier (Luo vessels) failure in holding the CPFs; (b) the lymphatic system displays a homeostatic role by removing pro-inflammatory agents into lymphatic nodes, delaying the aggravation of the disease [88], similarly with divergent channels, which act as a buffer between pathogens and internal organs, maintaining the disease in a latent stage;
2. Directional upward energetic flow;
3. Connections with large joints, passage through the chest or throat—areas characterized by a high density of lymph nodes, and convergence at the upper part of the body [33,54].

Given that targeting the lymphatic vessels was suggested as a potential therapeutic strategy in diseases with joint involvement [84,88,89], future integrative research may consider whether complementary approaches derived from Chinese medicine, including those associated with divergent channel therapy, could have any modulatory effects on lymphatic function in the SLE context.

The observed trend toward increasing zonulin levels across the study subgroups, coupled with the finding that group 1 (large-joint involvement) exhibited the lowest values, comparable to those of healthy controls, may indicate that large joints have a greater potential to retain CPFs than smaller joints. In SLE patients with large-joint involvement (signifying divergent channel latency phase), zonulin levels remain within the normal range, suggesting preserved gut barrier integrity and raising the hypothesis that CPFs may be differentially distributed and potentially sequestered within the joints. Nevertheless, this hypothesis derived from CCM remains speculative and warrants confirmation through longitudinal studies incorporating serial assessment of intestinal permeability, microbial translocation, patterns of articular and deep organ involvement, and accumulation of CPFs within joint tissues.

The direct correlation between the articular and skin involvements in both large and small joint involvement groups is highly significant from a Chinese medical perspective.

It may reflect that once the pathogen factor is diverted by divergent channels towards the joints and held latent in the articular area, there is a higher probability for activation of the sinew channels in the body’s attempt to eliminate the pathogen towards a more superficial energetic level (Wèi layer, which is occupied by the sinew channels), a process that may be associated with dermatological manifestations [32,33]. The functional coupling between divergent channels and sinew channels is possible due to the characteristic of divergent channels to support a protective layer (Wèi layer) by sending outward Yuán Qi from the constitutional layer (Yuán layer) [54].

### 4.2. Limitations of the Present Study

There are several limitations of the present study, one of the most important being the cross-sectional design of the study. Thus, the present findings should be interpreted as hypothesis-generating rather than as evidence of causality between gut leakage and SLE, and also between gut leakage and stages of pathogen invasion across the various energetic layers.

The Chinese concept of active pathogen sequestration in joints through divergent channel activation is also not supported by the present associative cross-sectional data.

Another major objective limitation is that the actual basis of the divergent channels and their relationship to the anatomical structures (e.g., lymphatic tissue), cellular and molecular aspects are still unknown and yet to be evaluated and better understood by conventional scientists. Therefore, the proposed biological correspondence between divergent channels and immunological pathways needs to be confirmed by further studies.

Another important limitation of the present study is the relatively small sample size. Moreover, the inclusion of multiple potential confounding factors that may influence plasma levels of zonulin and 1,3 BDG (including type of medication used, disease activity, dietary factors beyond the standardized fasting period, and metabolic variables) was not feasible from a statistical point of view, due to the limited sample. Consequently, future investigations, including larger cohorts and more comprehensive statistical analyses, are warranted to confirm the present findings and strengthen the reliability and translational significance of the results.

Additional limitations include the lack of newly diagnosed SLE patients and the fact that plasma zonulin levels do not exclusively reflect intestinal permeability, being influenced by several other factors.

There are also some recent concerns regarding potential analytical limitations of commercially available ELISA kits for zonulin measurement, which may lack sufficient sensitivity and may exhibit cross-reactivity with complement-related proteins [90]. Although median serum C3 levels were within the low-normal range, median C4 levels were reduced when compared with the commonly accepted reference [91]. Nevertheless, the reported analytical cross-reactivity has been described for complement C3, but not for C4 [90]. Thus, the reduced C4 values do not represent a directly demonstrated source of assay cross-reactivity. Hypocomplementemia in SLE subjects may falsely accentuate the differences between the SLE subjects and controls, but taking into account the C3 values in our SLE subjects, we estimate that this error was minimized. Further comparative studies evaluating zonulin measurements obtained using different commercially available ELISA kits are needed to determine the extent to which kit selection influences the results.

Therefore, in light of these considerations, our findings should be interpreted with caution, as the proposed interpretation remains hypothetical and requires further validation. 

## 5. Conclusions

SLE patients have higher levels of plasma zonulin and 1,3-BDG than healthy subjects, indicating impaired intestinal barrier and potential fungal translocation or gut proliferation.

Within the framework of CCM, plasma zonulin may be a candidate for the biomarkers of the latency phase in SLE (lower levels of plasma zonulin, higher degree of disease latency, and, therefore, higher involvement of divergent channels). However, larger studies with a longitudinal design are needed to confirm this hypothesis.

The observed increasing trend in plasma zonulin levels across the study groups—group 1 (large-joint involvement) < group 2 (small-joint involvement) < group 3 (no joint involvement)—should be interpreted with caution and further investigated through direct assessments of CPFs burden within affected joints to determine whether larger joints (due to their connection with divergent channels) might have a greater capacity to sequester CPFs than smaller joints.

A statistically significant correlation between articular and cutaneous involvement may, within the framework of CCM, indicate that the engagement of divergent channels is possibly associated with an increased likelihood of sinew channel activation as a potential mechanism of pathogen evacuation.

We emphasize that our integrative interpretations, based on both biomedicine and CCM, are speculative and should be regarded as hypothesis-generating. Our study should be considered a pilot study aimed at exploring the potential of this research direction, with the specific objective of identifying parameters worthy of investigation in order to formulate a clear and concise testable biomedical hypothesis for a subsequent, larger-scale study.

The causal relationship between increased intestinal permeability (“leaky gut”) and SLE, as well as its association with varying degrees of pathogenic progression across the bodily energetic layers, should be rigorously assessed in further prospective studies. Additionally, such investigations are needed to clarify the potential significance of articular involvement as a clinical landmark of latency of divergent channel pathology in SLE.

With a deeper understanding of the divergent channels system involvement in autoimmune diseases, such as SLE, new integrative therapeutic strategies with better results may evolve, especially in chronic unresponsive conditions.

## Figures and Tables

**Table 1:** Pathogenic factors associated with dysbiosis and leaky gut.

Climatic factors	high-humidity environment [34]
	heat exposure [35,36]
	seasonal climatic fluctuations [37]
Dietary factors	excessive fructose consumption [38]
	chronic high-fat diet [39,40]
Microbes	bacteria [41]
	viruses [42]
Environmental pollutants or xenobiotics	heavy metals [43]
	pesticides [44]
	micro- and nanoplastics [45]
	detergents [46]
	cosmetic pollutants [47]
	synthetic drugs (e.g., antibiotics, proton pump inhibitors, and anti-inflammatory agents) [48]
Psycho-emotional factors	psychological trauma [49]
	depression [50]
	anxiety [51]

**Table 2:** Energetic levels and their corresponding channels and anatomical structures [32,54,55,56].

Energetic Layer	Type of Qì (Potential Biomedical Correspondences)	Channels	Fluids and Anatomical Structures
Protective layer (Wèi layer)	Wèi Qì—Protective energy (immune system)	Sinew Channels	Jīn Fluids (impure) Muscles Skin
Nutritive layer (Yíng layer)	Yíng Qì—Nutritive energy (fluids—blood, lymph, plasma)	Primary Channels *	Jīn Fluids (pure) Yè Fluids Blood Xuè Flesh Internal organs (Zàng Fǔ) *
		Luo channels	Blood Xuè Blood vessels
Constitutional layer (*Yuán* layer)	Yuán Qì—constitutional energy (genetics) Jīng Qì—Essence (stem cells, genetics) Yuán Qì—functional aspect of essence	Extraordinary Channels Divergent Channels *	Marrow Bone, joints * Internal organs (Zàng Fǔ) *

Legend. * direct connection between channels and internal organs or anatomical structure.

**Table 3:** Demographic and anthropometric characteristics of patients with SLE and control group.

Variable	SLE Patients	Control Group	*p* Value
Sex (F/M)	32/1	22/2	NS (0.57)
Background (U/R)	20/13	19/5	NS (0.16)
Age (mean ± SD) [years]	43.21 ± 11.78	47.88 ± 12.45	NS (0.2)
BMI (mean ± SD) [kg/m^2^]	23.88 ± 4.04	25.87 ± 4.24	NS (0.08)

Note: There were no significant differences between the SLE patients group (*n* = 33) and the control (healthy individuals) group (*n* = 24) in terms of demographic and anthropometric variables: sex, age, body mass index (BMI), and background/residency (urban vs. rural). F = female, M = male, U = urban, R = rural, SD = standard deviation. Fisher’s exact test was used for categorical variables (sex, background); Student’s *t* test was employed for numerical variables (age, BMI).

**Table 4:** Clinical features of patients with SLE.

Variable	Median (IQR)
Disease duration since symptoms onset	9 (2–15)
Disease duration since diagnosis	7 (2–15)
SLEDAI score	4 (2–6)
SLICC/ACR Damage Index	2 (0–4)

Note: Results are presented as median (IQR).

**Table 5:** Biomarker profile and routine laboratory parameters of patients with SLE.

Variable	Median (IQR)
Zonulin (ng/mL)	4.709 (1.996–10.426)
1,3-BDG (pg/mL)	119.444 (82.746–277.66)
FGF21 (pg/mL)	281.38 (173,808–590.36)
WBC (×10^3^/mm^3^)	5.48 (4.77–7.21)
LYM (×10^3^/mm^3^)	1.03 (0.7–1.77)
NEU(×10^3^/mm^3^)	3.95 (2.68–5.26)
PLT (×10^3^/mm^3^)	260 (203–334)
Hb (g/dL)	12.6 (11.3–13.5)
Creatinine (mg/dL)	0.89 (0.8–0.98)
C3 serum level (mg/dL)	87.3 (72.01–106.47)
C4 serum level (mg/dL)	9.886 (6.211–18.995)
Anti-dsDNA ab (UI/mL)	34.4 (15.7–97)
ESR (mm/h)	16 (7–31)
CRP (mg/L)	4.51 (2.83–7.16)
24 h urine protein (mg/24 h)	140 (91.155–247.87)

Note: Results are presented as median (IQR); 1,3-BDG—1,3-beta-D-glucan, FGF21—fibroblast growth factor 21, WBC—white bloods cells, LYM—lymphocytes, NEU—neutrophiles, PLT—platelets, Hb—hemoglobin, C3—complement component 3, C4—complement component 4, Anti-dsDNA ab—anti-double stranded DNA antibodies, ESR—erythrocyte sedimentation rate, CRP—C reactive protein.

**Table 6:** Comparison between the study group and the control group regarding the three markers (Mann–Whitney test) estimated by means of the Mann–Whitney test.

Marker/Variable	Median (IQR) in SLE Patients	Median (IQR) in Control Group	Median Difference (95% Confidence Interval)	W Statistics	*p* Value
FGF21	281.38 (173.808–590.36)	538.2 (194.955–758.94)	145.442 (−52.160 to 380.002)	480	0.2
Zonulin	4.709 (1.996–10.426)	1.973 (1.371–3.05925)	−2.251 (−4.530 to −0.625)	220	0.004
1,3-BDG	119.444 (82.746–277.66)	77.893 (47.524–124.7495)	−39.902 (−81.398 to −10.192)	244	0.01

**Table 7:** The association between each of the three markers (Mann–Whitney test) and the type of involvement estimated by means of the Mann–Whitney test.

Marker/Variable	Median (IQR) for Patients with Cutaneous/Joint/Deep Organ Involvement	Median (IQR) for Patients Without Cutaneous/Joint/Deep Organ Involvement	Median Difference (95% Confidence Interval)	W Statistics	*p* Value
Cutaneous involvement (with/without: 20/13)					
FGF21	218.76 (160.9095–466.36)	428.62 (244.06–684.96)	129.227 (−79.334 to 403.58)	166	0.2
Zonulin	4.5295 (2.2575–6.681)	5.34 (1.531–11.759)	0.252 (−2.498 to 7.583)	135	0.9
1,3-BDG	118.173 (81.4515–200.9545)	130.574 (90.076–280.66)	8.362 (−41.884 to 100.6)	141.5	0.7
Joint involvement (with/without: 27/6)					
FGF21	244.06 (168.302–489.46)	583.11 (332.74–937.82)	288.062 (−92.304 to 772.260)	114	0.1
Zonulin	3.363 (1.816–6.1405)	15.436 (10.49–22.54575)	9.830 (2.872 to 20.847)	139	0.007
1,3-BDG	119.444 (80.157–226.523)	168.865 (93.6745–363.61)	25.76105 (−47.452 to 287.616)	95.5	0.5
Deep organ [renal/neurological involvement (with/without: 14/19)]					
FGF21	370.63 (173.672–590.77)	244.06 (174.787–583.11)	−47.086 (−267.494 to 135.624)	117	0.6
Zonulin	3.8565 (2.01225–6.23625)	5.102 (2.1085–10.5125)	1.034 (−1.691 to 5.667)	151	0.5
1,3-BDG	122.228 (91.0355–166.034)	116.902 (80.153–360.59)	11.400 (−36.698 to 181.082)	147	0.6

Legend. IQR—interquartile range.

**Table 8:** The association between zonulin and the type of joint involvement. Pairwise comparisons using the Mann–Whitney test with *p* value adjustment by the Bonferroni method.

Comparison	Median (IQR) in Patients with A	Median (IQR) in Patients with B	Median Difference (95% CI)	W Statistics	*p*-Value Bonferr.
A = small (*n* = 14) vs. B = large (*n* = 13)	5.2525 (2.9730–9.7515)	2.061 (1.672–4.350)	−2.9295 (−5.837 to 0.053)	52	0.37
A = small (*n* = 14) vs. B = without (*n* = 6)	5.2525 (2.9730–9.7515)	15.43600 (10.49000–22.54575)	−8.8015 (−20.327 to −0.374)	17	0.24
A = small (*n* = 14) vs. B = healthy controls (*n* = 24)	5.2525 (2.9730–9.7515)	1.97300 (1.37100–3.05925)	−3.289 (−5.817 to −0.932)	78	0.034
A = large (*n* = 13) vs. B = without (*n* = 6)	2.061 (1.672–4.350)	15.43600 (10.49000–22.54575)	−10.561 (−21.952 to −4.118)	6	0.013
A = large (*n* = 13) vs. B = healthy controls (*n* = 24)	2.061 (1.672–4.350)	1.97300 (1.37100–3.05925)	−0.2835 (−1.289 to 0.687)	138	1
A = without (*n* = 6) vs. B = healthy controls (*n* = 24)	15.43600 (10.49000–22.54575)	1.97300 (1.37100–3.05925)	−11.4715 (−22.037 to −7.136)	4	0.00024

Note: *p*-value Bonferr. = *p*-value with Bonferroni correction; 95% CI = 95% confidence interval.

**Table 9:** The association between cutaneous involvement and the type of joint involvement. Pairwise comparisons using Fisher’s exact test with *p* value adjustment by the Bonferroni method.

	Without Cut. Involv.	With Cut. Involv.	*p* Value	*p*-Value Bonferr.	Odds Ratio (95% CI)
Large ± small vs. small					
Large ± small joint involv.	4	9	0.6776	1	0.625 (0.072 to 4.805)
Small joint involv.	3	11			
Large ± small vs. without					
Large ± small joint involv.	4	9	0.01	0.03	Infinite (1.570 to infinite)
Without joint involv.	6	0			
Small vs. without					
Small joint involv	3	11	0.002	0.006	Infinite (2.375 to infinite)
Without joint involve.	6	0			

Note: cut. = cutaneous; involv. = involvement; *p*-value Bonferr. = *p*-value with Bonferroni correction; 95% CI = 95% confidence interval.

**Table 10:** The correlation between the three markers and disease duration by Spearman test with *p* value correction by the Bonferroni method.

Marker	Disease Duration	t_Statistics	CorreL. Coeff. (95% CI)	*p*-Value Bonferr.
FGF21	Disease duration since onset	5654.037	0.066 (−0.294 to 0.391)	1
FGF21	Disease duration since diagnosis	5360.862	0.104 (−0.248 to 0.432)	1
Zonulin	Disease duration since onset	7457.769	−0.246 (−0.544 to 0.106)	1
Zonulin	Disease duration since diagnosis	6881.078	−0.150 (−0.469 to 0.204)	1
BDG	Disease duration since onset	8014.249	−0.339 (−0.611 to 0.005)	0.32
BDG	Disease duration since diagnosis	8172.693	−0.366 (−0.630 to −0.026)	0.22

Note: CorreL. coeff. = correlation coefficient; 95% CI = 95% confidence interval; *p*-value Bonferr. = *p*-value with Bonferroni correction.

**Table 11:** The correlation between the three markers and SLEDAI by Spearman test with *p* value correction by the Bonferroni method.

Marker	t_Statistics	CorreL. Coeff. (95% CI)	*p*-Value Bonferr.
FGF21	6,531,033	−0.091 (−0.421 to 0.260)	1
Zonulin	5,361,724	0.104 (−0.248 to 0.432)	1
BDG	6,539,723	−0.093 (−0.423 to 0.259)	1

Note: CorreL. coeff. = correlation coefficient; 95% CI = 95% confidence interval; *p*-value Bonferr. = *p*-value with Bonferroni correction.

## Data Availability

The data presented in this study are available on request from the corresponding author. The data are not publicly available due to privacy restrictions.

## Abbreviations

The following abbreviations are used in this manuscript:

1,3-BDG	(1→3)-β-D-glucan
anti-dsDNA ab	anti-double-stranded DNA antibodies
BMMS	bone-marrow mesenchymal stem
C3	complement component 3
C4	complement component 4
CCM	Classical Chinese Medicine
CPFs	Chinese pathogenic factors
CRP	C-reactive protein
ELISA	enzyme-linked immunosorbent assay
ESR	erythrocyte sedimentation rate
FGF21	fibroblast growth factor 21
Hb	hemoglobin
LYM	lymphocytes
NEU	Neutrophiles
NA	not applicable
PLT	platelets
SLE	Systemic lupus erythematosus
SLEDAI	Systemic lupus erythematosus Disease Activity Index
SLICC/ACR	Systemic Lupus International Collaborating Clinics/American College of Rheumatology
WBC	White blood cells

## References

[1] Tselios K., Urowitz M.B. (2017). Cardiovascular and Pulmonary Manifestations of Systemic Lupus Erythematosus. Curr. Rheumatol. Rev..

[2] Zucchi D., Silvagni E., Elefante E., Signorini V., Cardelli C., Trentin F., Schilirò D., Cascarano G., Valevich A., Bortoluzzi A. (2023). Systemic Lupus Erythematosus: One Year in Review 2023. Clin. Exp. Rheumatol..

[3] Tang W., Khalili L., Gong R., Souvignier M., Wang X., Murray S., Chavez G.R., Nordmann-Gomes A., Geraldino-Pardilla L., Gartshteyn Y. (2025). Advanced Imaging in the Evaluation of Lupus Arthritis: A Systematic Literature Review and Meta-Analysis. Semin. Arthritis Rheum..

[4] Justiz Vaillant A.A., Goyal A., Varacallo M.A. (2026). Systemic Lupus Erythematosus. StatPearls [Internet].

[5] Dai X., Fan Y., Zhao X. (2025). Systemic Lupus Erythematosus: Updated Insights on the Pathogenesis, Diagnosis, Prevention and Therapeutics. Signal Transduct. Target. Ther..

[6] Guimarães de Oliveira D., Machado A., Lacerda P.C., Karakikla-Mitsakou Z., Vasconcelos C. (2025). Systemic Lupus Erythematosus and the Gut Microbiome: To Look Forward Is to Look within—A Systematic Review and Narrative Synthesis. Autoimmun. Rev..

[7] Charoensappakit A., Sae-Khow K., Leelahavanichkul A. (2022). Gut Barrier Damage and Gut Translocation of Pathogen Molecules in Lupus, an Impact of Innate Immunity (Macrophages and Neutrophils) in Autoimmune Disease. Int. J. Mol. Sci..

[8] López P., de Paz B., Rodríguez-Carrio J., Hevia A., Sánchez B., Margolles A., Suárez A. (2016). Th17 Responses and Natural IgM Antibodies Are Related to Gut Microbiota Composition in Systemic Lupus Erythematosus Patients. Sci. Rep..

[9] Wendling D., Prati C. (2018). Spondyloarthritis: An Expanding Cast of Cellular Actors. Jt. Bone Spine.

[10] O’Keeffe K.M., Wilk M.M., Leech J.M., Murphy A.G., Laabei M., Monk I.R., Massey R.C., Lindsay J.A., Foster T.J., Geoghegan J.A. (2015). Manipulation of Autophagy in Phagocytes Facilitates Staphylococcus Aureus Bloodstream Infection. Infect. Immun..

[11] Berthelot J.-M., Claudepierre P. (2016). Trafficking of Antigens from Gut to Sacroiliac Joints and Spine in Reactive Arthritis and Spondyloarthropathies: Mainly through Lymphatics?. Jt. Bone Spine.

[12] Berthelot J.-M., Wendling D. (2020). Translocation of Dead or Alive Bacteria from Mucosa to Joints and Epiphyseal Bone-Marrow: Facts and Hypotheses. Jt. Bone Spine.

[13] Ahamefula Osibe D., Lei S., Wang B., Jin C., Fang W. (2020). Cell Wall Polysaccharides from Pathogenic Fungi for Diagnosis of Fungal Infectious Disease. Mycoses.

[14] Smadu S.G., Tetradov S.C., Ene L., Oprisan C., Lazăr D.Ș., Florescu S.A. (2026). Diagnostic Biomarkers for Invasive Candidiasis: A Clinician-Oriented Review. J. Fungi.

[15] Cheibchalard T., Leelahavanichkul A., Chatthanathon P., Klankeo P., Hirankarn N., Somboonna N. (2024). Fungal Microbiome in Gut of Systemic Lupus Erythematosus (SLE)-Prone Mice (Pristane and FCGRIIb Deficiency), a Possible Impact of Fungi in Lupus. PLoS ONE.

[16] Thim-Uam A., Surawut S., Issara-Amphorn J., Jaroonwitchawan T., Hiengrach P., Chatthanathon P., Wilantho A., Somboonna N., Palaga T., Pisitkun P. (2020). Leaky-Gut Enhanced Lupus Progression in the Fc Gamma Receptor-IIb Deficient and Pristane-Induced Mouse Models of Lupus. Sci. Rep..

[17] Saithong S., Saisorn W., Visitchanakun P., Sae-Khow K., Chiewchengchol D., Leelahavanichkul A. (2021). A Synergy Between Endotoxin and (1→3)-Beta-D-Glucan Enhanced Neutrophil Extracellular Traps in Candida Administered Dextran Sulfate Solution Induced Colitis in FcGRIIB-/- Lupus Mice, an Impact of Intestinal Fungi in Lupus. J. Inflamm. Res..

[18] Issara-Amphorn J., Surawut S., Worasilchai N., Thim-uam A., Finkelman M., Chindamporn A., Palaga T., Hirankarn N., Pisitkun P., Leelahavanichkul A. (2018). The Synergy of Endotoxin and (1→3)-β-D-Glucan, from Gut Translocation, Worsens Sepsis Severity in a Lupus Model of Fc Gamma Receptor IIb-Deficient Mice. J. Innate Immun..

[19] Liu H.-M., Chang Z.-Y., Yang C.-W., Chang H.-H., Lee T.-Y. (2023). Farnesoid X Receptor Agonist GW4064 Protects Lipopolysaccharide-Induced Intestinal Epithelial Barrier Function and Colorectal Tumorigenesis Signaling through the αKlotho/βKlotho/FGFs Pathways in Mice. Int. J. Mol. Sci..

[20] Chu C., Li T., Yu L., Li Y., Li M., Guo M., Zhao J., Zhai Q., Tian F., Chen W. (2023). A Low-Protein, High-Carbohydrate Diet Exerts a Neuroprotective Effect on Mice with 1-Methyl-4-Phenyl-1,2,3,6-Tetrahydropyridine-Induced Parkinson’s Disease by Regulating the Microbiota-Metabolite-Brain Axis and Fibroblast Growth Factor 21. J. Agric. Food Chem..

[21] Lin D., Sun Q., Liu Z., Pan J., Zhu J., Wang S., Jia S., Zheng M., Li X., Gong F. (2023). Gut Microbiota and Bile Acids Partially Mediate the Improvement of Fibroblast Growth Factor 21 on Methionine-Choline-Deficient Diet-Induced Non-Alcoholic Fatty Liver Disease Mice. Free Radic. Biol. Med..

[22] Chen Q., Cheng W., Zhang J., Chi C., Lin M., He C., Liao Z., Gong F. (2024). Fibroblast Growth Factor 21 Improves Insulin Sensitivity by Modulating the Bile Acid-Gut Microbiota Axis in Type II Diabetic Mice. Free Radic. Biol. Med..

[23] Zou Y., Wang D., Sun W., Wu Q., Liu S., Ren Z., Li Y., Zhao T., Li Z., Li X. (2024). Fibroblast Growth Factor 21 Mitigates Lupus Nephritis Progression via the FGF21/Irgm 1/NLRP3 Inflammasome Pathway. Int. Immunopharmacol..

[24] Halfon M., Memon A.A., Hedelius A., Pascual M., Sundquist K., Ribi C. (2025). Lower Circulating Mitochondrial DNA and Increased Mitokines Suggest Significant Mitochondrial Dysfunction in Systemic Lupus Erythematosus with Renal Involvement. Lupus Sci. Med..

[25] Tajik N., Frech M., Schulz O., Schälter F., Lucas S., Azizov V., Dürholz K., Steffen F., Omata Y., Rings A. (2020). Targeting Zonulin and Intestinal Epithelial Barrier Function to Prevent Onset of Arthritis. Nat. Commun..

[26] Ilie C., Stoian I.A., Gaman L.E., Gro L., Rodica A., Gîlc M. (2025). Increased Intestinal Permeability and Articular Involvement in Systemic Lupus Erythematosus Patients—A Mutually Exclusive Relationship?. Curr. Issues Mol. Biol..

[27] Lefferts A.R., Norman E., Claypool D.J., Kantheti U., Kuhn K.A. (2022). Cytokine Competent Gut-Joint Migratory T Cells Contribute to Inflammation in the Joint. Front. Immunol..

[28] Salmi M., Jalkanen S. (2005). Lymphocyte Homing to the Gut: Attraction, Adhesion, and Commitment. Immunol. Rev..

[29] Stagg A.J., Kamm M.A., Knight S.C. (2002). Intestinal Dendritic Cells Increase T Cell Expression of Alpha4beta7 Integrin. Eur. J. Immunol..

[30] Iwata M., Hirakiyama A., Eshima Y., Kagechika H., Kato C., Song S.-Y. (2004). Retinoic Acid Imprints Gut-Homing Specificity on T Cells. Immunity.

[31] Svensson M., Marsal J., Ericsson A., Carramolino L., Brodén T., Márquez G., Agace W.W. (2002). CCL25 Mediates the Localization of Recently Activated CD8αβ^+^ Lymphocytes to the Small-Intestinal Mucosa. J. Clin. Investig..

[32] Cecil-Sterman A. (2012). Advanced Acupuncture: A Clinic Manual. Protocols for the Complement Channels of the Complete Acupuncture System: The Sinew, Luo, Divergent and Eight Extraordinary Channels.

[33] Yuen J. (2004). Channel Systems of Chinese Medicine—Divergent Meridians.

[34] Wang Y., Zhuang K., Yi Q., Wu Y., Luo Y., Ouyang Y., Li L., Li C., Luo H. (2025). High Humidity Environment Increases FBG by Impairing the Intestinal Barrier. Front. Immunol..

[35] Vliora M., McCormick J.J., King K.E., Gkiata P., Kaltsatou A., Flouris A.D., Kenny G.P. (2025). Brief Extreme Passive Heat Exposure Leads to Elevated Biomarkers of Systemic Inflammation and Acute Kidney Injury in Older vs Young Adults. Eur. J. Appl. Physiol..

[36] Ghulam Mohyuddin S., Khan I., Zada A., Qamar A., Arbab A.A.I., Ma X.-B., Yu Z.-C., Liu X.-X., Yong Y.-H., Ju X.H. (2022). Influence of Heat Stress on Intestinal Epithelial Barrier Function, Tight Junction Protein, and Immune and Reproductive Physiology. BioMed. Res. Int..

[37] Wang Q., Lu Q., Tang Y., Li Q., Gao P., Pang S., Zhang W., Nie C., Niu J., Ma X. (2025). Integrated 16S rDNA-Seq and Metabolomics Reveal Seasonal Dynamics of Gut Microbial-SCFA-Immune Crosstalk in Diarrheic Calves. Front. Vet. Sci..

[38] Cho Y.-E., Kim D.-K., Seo W., Gao B., Yoo S.-H., Song B.-J. (2021). Fructose Promotes Leaky Gut, Endotoxemia, and Liver Fibrosis Through Ethanol-Inducible Cytochrome P450-2E1-Mediated Oxidative and Nitrative Stress. Hepatology.

[39] Miranda C.S., Santana-Oliveira D.A., Vasques-Monteiro I.L., Dantas-Miranda N.S., de Oliveira Glauser J.S., Silva-Veiga F.M., Souza-Mello V. (2024). Time-Dependent Impact of a High-Fat Diet on the Intestinal Barrier of Male Mice. World J. Methodol..

[40] Rodrigues P.B., Dátilo M.N., Sant’ANa M.R., Nogueira G.A.d.S., Marin R.M., Nakandakari S.C.B.R., de Moura L.P., da Silva A.S.R., Ropelle E.R., Pauli J.R. (2023). The Early Impact of Diets Enriched with Saturated and Unsaturated Fatty Acids on Intestinal Inflammation and Tight Junctions. J. Nutr. Biochem..

[41] Omarova S., Awad K., Moos V., Püning C., Gölz G., Schulzke J.-D., Bücker R. (2023). Intestinal Barrier in Post-Campylobacter Jejuni Irritable Bowel Syndrome. Biomolecules.

[42] Almanaa T.N., Al-Kuraishy H.M., Al-Gareeb A.I., Abdelnaby M.A., Alexiou A., Papadakis M., Abo-ElFetoh I.E., Batiha G.E.-S. (2026). SARS-CoV-2 Infection and Gut-Lung Axis: The Potential Role of Rifaximin. Inflammopharmacology.

[43] Kaur R., Rawal R. (2023). Influence of Heavy Metal Exposure on Gut Microbiota: Recent Advances. J. Biochem. Mol. Toxicol..

[44] Osa-Subtil I., Romero-Rosales T., Dios-Duarte M.J. (2026). Impact of Pesticide Use on Gut Microbiota and Health: A Systematic Review of Findings in Both Humans and Animal Models. J. Xenobiotics.

[45] Pacher-Deutsch C., Schweighofer N., Hanemaaijer M., Marut W., Žukauskaitė K., Horvath A., Stadlbauer V. (2025). The Microplastic-Crisis: Role of Bacteria in Fighting Microplastic-Effects in the Digestive System. Environ. Pollut..

[46] Ogulur I., Pat Y., Aydin T., Yazici D., Rückert B., Peng Y., Kim J., Radzikowska U., Westermann P., Sokolowska M. (2023). Gut Epithelial Barrier Damage Caused by Dishwasher Detergents and Rinse Aids. J. Allergy Clin. Immunol..

[47] Kiran N.S., Yashaswini C., Chatterjee A. (2024). Noxious Ramifications of Cosmetic Pollutants on Gastrointestinal Microbiome: A Pathway to Neurological Disorders. Life Sci..

[48] Totleben L., Thomas J., Austin D. (2025). Drug-Mediated Disruption of the Aging Gut Microbiota and Mucosal Immune System. Front. Aging.

[49] Ciydem E., Arslan S. (2026). Psychological Trauma and Gut Health: Unlocking the Potential of Dietary Interventions to Modulate the Gut-Brain Axis. J. Psychosoc. Nurs. Ment. Health Serv..

[50] Su Y., Xia Y. (2026). Gut Microbiota Dysbiosis and Depression: Bidirectional Interactions, Mediating Pathways, and Microecological Therapeutics. Curr. Res. Food Sci..

[51] Bjørklund G., Butnariu M., Dadar M., Semenova Y. (2025). The Gut Microbiota-Anxiety Connection: Evidence, Mechanisms, and Therapeutic Strategies. Curr. Med. Chem..

[52] Ma L., Morel L. (2022). Loss of Gut Barrier Integrity in Lupus. Front. Immunol..

[53] Manfredo Vieira S., Hiltensperger M., Kumar V., Zegarra-Ruiz D., Dehner C., Khan N., Costa F.R.C., Tiniakou E., Greiling T., Ruff W. (2018). Translocation of a Gut Pathobiont Drives Autoimmunity in Mice and Humans. Science.

[54] Montakab H. (2014). Acupuncture Point and Channel Energetics. Bridging the Gap.

[55] Qiu J. (2015). When the East Meets the West: The Future of Traditional Chinese Medicine in the 21st Century. Natl. Sci. Rev..

[56] Ju L., Jiang J., Jin Y., Armand J.-P., Charron D. (2023). Modern Immunology Is Crucial to Revealing the Biological Mechanisms of Traditional Chinese Medicine. J. Tradit. Chin. Med. Sci..

[57] Chrisman C., Xie H. (2013). The Jing Luo Network: An Overview of Channels and Collaterals and Their Clinical Applications. Am. J. Tradit. Chin. Vet. Med..

[58] Nguyen K.D., Fentress S.J., Qiu Y., Yun K., Cox J.S., Chawla A. (2013). Circadian Gene Bmal1 Regulates Diurnal Oscillations of Ly6C(Hi) Inflammatory Monocytes. Science.

[59] Narasimamurthy R., Hatori M., Nayak S.K., Liu F., Panda S., Verma I.M. (2012). Circadian Clock Protein Cryptochrome Regulates the Expression of Proinflammatory Cytokines. Proc. Natl. Acad. Sci. USA.

[60] Unschuld P.U. (2016). Huang Di Nei Jing Ling Shu.

[61] Hand L.E., Hopwood T.W., Dickson S.H., Walker A.L., Loudon A.S.I., Ray D.W., Bechtold D.A., Gibbs J.E. (2016). The Circadian Clock Regulates Inflammatory Arthritis. FASEB J..

[62] Aringer M., Costenbader K., Daikh D., Brinks R., Mosca M., Ramsey-Goldman R., Smolen J.S., Wofsy D., Boumpas D.T., Kamen D.L. (2019). 2019 European League Against Rheumatism/American College of Rheumatology Classification Criteria for Systemic Lupus Erythematosus. Arthritis Rheumatol..

[63] Thanou A., James J.A., Arriens C., Aberle T., Chakravarty E., Rawdon J., Stavrakis S., Merrill J.T., Askanase A. (2019). Scoring Systemic Lupus Erythematosus (SLE) Disease Activity with Simple, Rapid Outcome Measures. Lupus Sci. Med..

[64] Gladman D.D., Urowitz M.B. (1999). The SLICC/ACR Damage Index: Progress Report and Experience in the Field. Lupus.

[65] Chu X., Li S., Wang Y., Guo D., Zhao N., Han Y., Xing Q. (2025). Alteration in Gut Microbiota Accompanied by Increased Intestinal Permeability and Tfh/Tfr Imbalance in Patients with Active SLE. Front. Cell. Infect. Microbiol..

[66] Leloix A., Ropert-Bouchet M., Cavillon T., Island M.-L., Loreal O., Guggenbuhl P., Robin F. (2026). Investigating Synovial Trace Elements as Diagnostic Markers in Acute Septic Arthritis: An Exploratory Study. Clin. Rheumatol..

[67] Krachler M., Domej W., Irgolic K.J. (2000). Concentrations of Trace Elements in Osteoarthritic Knee-Joint Effusions. Biol. Trace Elem. Res..

[68] Li Z., Zheng Y., Maimaiti Z., Fu J., Yang F., Li Z.-Y., Shi Y., Hao L.-B., Chen J.-Y., Xu C. (2024). Identification and Analysis of Microplastics in Human Lower Limb Joints. J. Hazard. Mater..

[69] Yang Q., Peng Y., Wu X., Cao X., Zhang P., Liang Z., Zhang J., Zhang Y., Gao P., Fu Y. (2025). Microplastics in Human Skeletal Tissues: Presence, Distribution and Health Implications. Environ. Int..

[70] Pamphlett R., Kum Jew S. (2019). Mercury Is Taken Up Selectively by Cells Involved in Joint, Bone, and Connective Tissue Disorders. Front. Med..

[71] Stull C., Sprow G., Werth V.P. (2023). Cutaneous Involvement in Systemic Lupus Erythematous: A Review for the Rheumatologist. J. Rheumatol..

[72] Psarras A., Wittmann M., Vital E.M. (2022). Emerging Concepts of Type I Interferons in SLE Pathogenesis and Therapy. Nat. Rev. Rheumatol..

[73] Blanco A.M., Bertucci J.I., Unniappan S. (2020). FGF21 Mimics a Fasting-Induced Metabolic State and Increases Appetite in Zebrafish. Sci. Rep..

[74] Prida E., Álvarez-Delgado S., Pérez-Lois R., Soto-Tielas M., Estany-Gestal A., Fernø J., Seoane L.M., Quiñones M., Al-Massadi O. (2022). Liver Brain Interactions: Focus on FGF21 a Systematic Review. Int. J. Mol. Sci..

[75] Salgado J.V., Goes M.A., Filho N.S. (2021). FGF21 and Chronic Kidney Disease. Metab.-Clin. Exp..

[76] Yu Y., Li S., Liu Y., Tian G., Yuan Q., Bai F., Wang W., Zhang Z., Ren G., Zhang Y. (2015). Fibroblast Growth Factor 21 (FGF21) Ameliorates Collagen-Induced Arthritis through Modulating Oxidative Stress and Suppressing Nuclear Factor-Kappa B Pathway. Int. Immunopharmacol..

[77] Chace C. (2018). Fantasy Maps and How to Use Them: Rooting Channel Divergence Theory in Palpatory Experience. J. Chin. Med..

[78] Phan T. (2017). American Chinese Medicine. Ph.D. Thesis.

[79] Pisetsky D.S. (2016). Anti-DNA Antibodies—Quintessential Biomarkers of SLE. Nat. Rev. Rheumatol..

[80] Tsokos G.C. (2011). Systemic Lupus Erythematosus. N. Engl. J. Med..

[81] Ghodke-Puranik Y., Olferiev M., Crow M.K. (2024). Systemic Lupus Erythematosus Genetics: Insights into Pathogenesis and Implications for Therapy. Nat. Rev. Rheumatol..

[82] Nelson M., Murthy S., Maddipatla S., Shenoy S., Ponder L., Chandrakasan S., Kugathasan S., Cutler D., Prahalad S. (2026). Genetic Determinants of Childhood Onset Systemic Lupus Erythematosus. Lupus Sci. Med..

[83] Popescu M., Tripon M.A., Lupșan A.F., Bungărdean D., Crecan C.M., Musteata M., Pașca P.M., Mârza S.M., Purdoiu R.C., Papuc I. (2025). Sentinel Equines in Anthropogenic Landscapes: Bioaccumulation of Heavy Metals and Hematological Biomarkers as Indicators of Environmental Contamination. Toxics.

[84] Smith M.D. (2011). The Normal Synovium. Open Rheumatol. J..

[85] Xu H., Edwards J., Banerji S., Prevo R., Jackson D.G., Athanasou N.A. (2003). Distribution of Lymphatic Vessels in Normal and Arthritic Human Synovial Tissues. Ann. Rheum. Dis..

[86] Cao M., Ong M.T.Y., Yung P.S.H., Tuan R.S., Jiang Y. (2022). Role of Synovial Lymphatic Function in Osteoarthritis. Osteoarthr. Cartil..

[87] Zhou Q., Wood R., Schwarz E.M., Wang Y.-J., Xing L. (2010). Near Infrared Lymphatic Imaging Demonstrates the Dynamics of Lymph Flow and Lymphangiogenesis during the Acute vs. chronic phases arthritis mice. Arthritis Rheum..

[88] Luo Y., Song J., Zheng S., Sun J., Liu G., Xiao Z., Opoku M., Arthur Vithran D.T., Zhou J., Xiao W. (2026). The Role of the Lymphatic System in Musculoskeletal System Health and Disease: Research Progress and Future Directions. Bone Res..

[89] Lin X., Bell R.D., Catheline S.E., Takano T., McDavid A., Jonason J.H., Schwarz E.M., Xing L. (2023). Targeting Synovial Lymphatic Function as a Novel Therapeutic Intervention for Age-Related Osteoarthritis in Mice. Arthritis Rheumatol..

[90] Ajamian M., Steer D., Rosella G., Gibson P.R. (2019). Serum Zonulin as a Marker of Intestinal Mucosal Barrier Function: May Not Be What It Seems. PLoS ONE.

[91] Ritchie R.F., Palomaki G.E., Neveux L.M., Navolotskaia O., Ledue T.B., Craig W.Y. (2004). Reference Distributions for Complement Proteins C3 and C4: A Practical, Simple and Clinically Relevant Approach in a Large Cohort. J. Clin. Lab. Anal..

